# Challenges in standardization of blood pressure measurement at the population level

**DOI:** 10.1186/s12874-015-0020-3

**Published:** 2015-04-10

**Authors:** Hanna Tolonen, Päivikki Koponen, Androniki Naska, Satu Männistö, Grazyna Broda, Tarja Palosaari, Kari Kuulasmaa, for the EHES Pilot Project

**Affiliations:** Department of Health, National Institute for Health and Welfare, Helsinki, Finland; Hellenic Health Foundation, Athens, Greece and Department of Hygiene, Epidemiology and Medical Statistics, University of Athens Medical School, Athens, Greece; The Cardinal Stefan Wyszynski Institute of Cardiology, Warsaw, Poland

**Keywords:** Blood pressure, Surveillance, Methodology

## Abstract

**Background:**

Accurate blood pressure measurements are needed in clinical practice, intervention studies and health examination surveys. Blood pressure measurements are sensitive: their accuracy can be affected by measurement environment, behaviour of the subject, measurement procedures, devices used for the measurement and the observer. To minimize errors in blood pressure measurement, a standardized measurement protocol is needed.

**Methods:**

The European Health Examination Survey (EHES) Pilot project was conducted in 2009–2012. A pilot health examination survey was conducted in 12 countries using a standardized protocol. The measurement protocols used in each survey, training provided for the measurers, measurement data, and observations during site visits were collected and evaluated to assess the level of standardization.

**Results:**

The EHES measurement protocol for blood pressure was followed accurately in all 12 pilot surveys. Most of the surveys succeeded in organizing a quiet and comfortable measurement environment, and staff instructed survey participants appropriately before examination visits. In all surveys, blood pressure was measured three times, from the right arm in a sitting posture. The biggest variation was in the device used for the blood pressure measurement.

**Conclusions:**

It is possible to reach a high level of standardization for blood pressure measurements across countries and over time. A detailed, standardized measurement protocol, and adequate training and monitoring during the fieldwork and centrally organized quality assessment of the data are needed. The recent EU regulation banning the sale of mercury sphygmomanometer in European Union Member States has set new challenges for the standardization of measurement devices since the validity of oscillometric measurements is device-specific and performance of aneroid devices depends very much on calibration.

## Background

High blood pressure is a major risk factor for vascular disease, such as ischaemic heart disease and stroke. High blood pressure can be lowered by lifestyle changes and medical treatment. Small changes in the average blood pressure values of the population may be of considerable importance to public health [1]. Accuracy of blood pressure measurement is important in clinical practice, intervention studies and health examination surveys. Measured blood pressure is influenced by the measurement environment, behavior of the subject, measurement protocol, device used for the measurement, and the observer.

Environmental factors such as the temperature of the room in which the blood pressure is measured and possible disturbance (eg. traffic noise, telephone ringing, and people entering the room) during the measurement may affect the outcome [2]. Activities such as strenuous physical exercise, smoking, heavy meals or drinking of coffee, tea or alcohol, or an uncomfortably full bladder are known to alter blood pressure [3,4]. The measurement protocol, the detailed instructions for how the blood pressure should be measured, has a significant role in the accuracy of the blood pressure results. Resting time before measurements, the arm used for the measurement, posture of the subject (sitting or supine) and the arm during the measurement, support of the back and feet, crossed legs, placement of the cuff over the clothing or on the bare arm, and talking during the measurement each affect the outcome [3-12].

The simple mercury sphygmomanometer has long been considered as a ‘gold standard’ for blood pressure measurement. Commonly used alternatives for the mercury sphygmomanometer are oscillometric devices, which should prevent the observer error present when the mercury sphygmomanometer is used. Validation protocols have been defined to ensure that oscillometric devices measure accurately in comparison with the mercury sphygmomanometer. It is important to ensure that devices used have passed the validation against at least one of the following: the British Hypertension Society protocol [13], International protocol [14] or Association for the Advancement of Medical Instrumentation (AAMI) protocol [15]. Regardless of the type of blood pressure measurement device, selection of the correct cuff size is important [11] and when the auscultation method is used, the side of the stethoscope used may have an effect on the outcome [16,17]. For all device types, mercury and aneroid sphygmomanometers, and oscillometric devices, calibration error may also bias the results [18,19].

Common observer errors in the auscultation method are systematic error, terminal digit preference and observer prejudice or bias [20]. Systematic error occurs in auscultation method when the observer does not hear well enough, or has slow reactions to auditory and visual cues. There may also be problems in the interpretation of the Korotkoff sounds [21]. Theoretically it is expected that terminal digits of the blood pressure readings are evenly distributed. When mercury sphygmomanometers are used, there should be equal proportions of terminal digits of 0, 2, 4, 6, and 8 and for oscillometric devices terminal digits from 0 to 9. Terminal digit preference is a common problem in both clinical practice and in epidemiological studies. Often the preference is observed for a terminal digit of zero. Some observers also have a tendency to avoid or prefer certain blood pressure readings or, when sequential readings are taken, to provide identical readings [22-24].

Table 1 summarizes reported effects of different factors influencing observed systolic and diastolic blood pressure values. The effects vary from 1–2 mmHg up-to 20–50 mmHg. These can have a large effect on treatment decisions. They can also bias substantially population estimates of blood pressure levels and hypertension prevalence derived from health examination surveys. Using data from Canadian and UK surveys, it has been shown that overestimation of systolic blood pressure by 3 and 5 mmHg increases the number of persons classified as hypertensive by 24% and 43% respectively; underestimation by the same amount misses 19% and 30% of persons with hypertension [25]. Without proper standardization of the measurements, true population level changes may be mixed up with the effects of measurement error.

**Table 1:** **Reported effect of different factors of observed blood pressure levels**

**Related to**	**Factor**	**Effect on systolic blood pressure (mmHg)**	**Effect on diastolic blood pressure (mmHg)**
Environment in which measurement is conducted	Cold room vs. comfortable room temperature [2]	⇑ 14 mmHg	⇑ 15 mmHg
Person being measured	Full bladder [3,4]	⇑ 10–15 mmHg, in case of uncomfortably distended bladder	⇑ 10 mmHg, in case of uncomfortably distended bladder ⇑ 40 mmHg
		⇑ 50 mmHg	
	Heavy physical exercise before measurement [3]	⇓ 18–20 mmHg	⇓ 7–9 mmHg
	Heavy meal before measurement [3]	⇓ 20 mmHg	⇓ 20 mmHg
	Smoking before measurement [3]	⇑ 10 mmHg	⇑ 8 mmHg
Measurement procedure	Not resting at least 5 min before measurement [5]	⇑ 10–20 mmHg	⇑ 14 mmHg
	Left arm vs. right arm [6]	⇓ 1–3 mmHg	⇑ 1 mmHg
	Supine vs. sitting [7]	⇑ 3–10 mmHg	⇑ 1–5 mmHg
	Back/feet unsupported vs. supported [4]	⇑ 5–15 mmHg	⇑ 6 mmHg
	Arm unsupported during the measurement vs. arm supported [4,11]	⇑ 1–7 mmHg	⇑ 5–11 mmHg
	Legs crossed vs. not crossed [8]	⇑ 5–8 mmHg	⇑ 3–5 mmHg
	Talking during the measurement vs. silent [4,12]	⇑ 17 mmHg	⇑ 13 mmHg
	Arm below heart level vs. arm at the heart level [9-11]	⇑ 10 mmHg	⇑ 10 mmHg
	Cuff over clothing vs. cuff on bare arm [4]	⇑ 5 mmHg	not reported
Device	Cuff too large [11]	⇓ 10–30 mmHg	⇓ 10–30 mmHg
	Cuff too small [11]	⇑ 3–12 mmHg, in obese persons	⇑ 2–8 mmHg, in obese persons
		⇑ 30 mmHg	⇑ 30 mmHg
	Diaphragm of stethoscope vs. bell (auscultation method used) [16,17]	⇓ 0–2 mmHg	⇓ 0–2 mmHg
	Calibration error [18,19]	0–5 mmHg	0–5 mmH

⇑ Arrow upwards: increases observed blood pressure level; ⇓ arrow down wards: decreases observed blood pressure level.

To minimize errors in blood pressure measurement, standardized measurement protocols have been proposed, for example by the European Society of Hypertension [11] and the American Heart Association [26]. These protocols are for clinical measurement of blood pressure. There are also international blood pressure measurement protocols for epidemiological studies, proposed by World Health Organization (WHO) MONICA Project [27], WHO in the Cardiovascular Survey Methods [28] and the European Health Examination Survey (EHES) Project [29].

The aim of this paper is to evaluate the level of standardization of blood pressure measurement in the EHES Pilot Project.

## Methods

### European health examination survey

EHES is a collaboration for standardizing national health examination surveys in Europe. The EHES Pilot Project was conducted in 2009–2012. The EHES Reference Centre (EHES RC) was established to coordinate the activities. The EHES RC prepared the European level standardized health examination survey (HES) protocol; provided support to the countries in planning and preparing their national HESs; organized European level training and provided training materials; organized external quality assessment; and evaluated the survey outcomes.

Twelve countries (Czech Republic, Finland, Germany, Greece, Italy, Malta, the Netherlands, Norway, Poland, Portugal, Slovakia and UK/England) planned and prepared for their national HES and conducted a pilot survey to test the feasibility of their survey protocol [30]. In four pilot countries (Germany, Italy, Netherlands and UK/England), a full-size national HES was on-going. In these countries, the aim was to evaluate how the EHES protocols could be incorporated into the on-going HES.

These pilot surveys, consisting in total of 4127 participants aged 25–64 years, were conducted in geographically defined populations. The pilot surveys were not nationally representative. Each survey was conducted by a local survey team after approval from the national/regional ethics committee (Appendix 1). Written informed consent was obtained from the survey participants.

### Standardized protocol

The EHES Manual provides standardized protocols for conducting HESs, including planning, running the fieldwork and reporting [31,32]. The blood pressure measurement protocol is based on three sequential measurements, one minute apart, from the right arm in a sitting posture. The survey participant should be sitting on a chair with the participant’s feet firmly on the floor and not crossed, and with their back supported by the chair. The participant’s arm should be resting, palm up, on a desk, table or arm rest of the chair so that the antecubital fossa is at the heart level. Before measurement, the participant is asked to remove all the clothing that might prevent the proper placement of the cuff on bare skin or be otherwise restricting around the upper arm, and to sit quietly for five minutes. The mid upper arm circumference is measured and the correct cuff size is selected from three to four available cuffs. If a simple mercury sphygmomanometer is used, the bell of the stethoscope is used for auscultation [29].

The EHES Manual has a template for a recording form for the blood pressure measurements. This form includes activities and behaviours of the participant before measurement, reasons for deviations from the standardized protocol, information about the measurement environment and measurement results [32].

### Training

The national trainers participated in the three day European level training seminar organized by the EHES RC. The key points of the measurements were discussed and practical training on real subjects was conducted. During the training, both mercury sphygmomanometer and oscillometric devices were used. The training included details about the posture of the subject during the measurement, how the arm circumference should be measured and the correct cuff size selected, instruction to be given to the participant (not to move or talk during the measurement), and how actual measurements should be taken. For the mercury sphygmomanometer, measurement training also included determination of the peak inflation level, the deflation rate to be used and determination of Korotkoff sounds (Phase I and Phase V). Training materials (presentations and videos) are available at the EHES web site [33].

The joint training session was followed by national training of the fieldwork personnel in each country. The duration and contents of the training varied both by country and the previous experience of the fieldwork staff.

### Evaluation

#### Site visits

The success of the standardization was assessed during site visits by members of the EHES RC to observe the fieldwork in each country. The measurement environment (room temperature, disturbing noises, lighting, adequacy of the table and chair for the measurement), interaction between the subject and the measurer, the measurement procedure and equipment used for the blood pressure measurement (brand and type of the device, number of cuffs available) were recorded. Observations were documented in site visit reports and feedback to local survey organizers and fieldwork teams was provided.

#### Retrospective data assessment

A retrospective quality assessment of the measurements was conducted, based on national survey protocols and collected data. This assessment included comparison of the national survey protocols with the EHES protocol, evaluation of the proportion of missing data for each data item, proportion of identical sequential readings and the proportion of terminal digits. High proportions of identical sequential readings or terminal digit preference is an indicator of problems with the measurements, usually reflecting the lack of proper training.

## Results

Most of the EHES pilot surveys were conducted in a clinical setting, either in the facilities of the local health care system or in temporary clinics set up specifically for the survey. In one survey, the measurements were conducted in the participants’ home and another survey used a mobile examination unit. From the measurement environment, the room temperature was measured and recorded routinely in nine surveys. The average room temperature varied from 19.4°C to 25.0°C, with relatively small variation within survey (Table 2). In some countries, occasional traffic noise outside the examination room or disturbances such as other personnel entering the room or a phone ringing during the measurements was observed during the site visits.

**Table 2:** **Blood pressure measurement protocols used in EHES pilot surveys**

**Pilot survey**	**Number of participants**	**Survey period**	**Mean room temperature °C (min, max)**	**Arm used**	**Posture of the subject during the measurement**	**Device type**	**Device model**	**Used cuff sizes (recommended arm circumference)**	**Side of stethoscope when auscultation method used**
A	123	May-Jun 2011	22.3 (21,24)	Right	Sitting	Mercury sphygmomanometer	Riester diplomat presometer	22–32 cm and 33–41 cm	Bell
B	111	Apr-Jul 2011	25.0 (18,33)	Right^1,2^	Sitting^3,4^	Mercury sphygmomanometer	Riester diplomat presometer	17–26 cm, 24–32 cm, 32–48 cm	Diaphragm
C	393	Oct 2010-Jan 2011	22.8 (21,26)	Right	Sitting	Mercury sphygmomanometer	Riva Rocci	≤34 cm, > 34 cm	Bell
D	183	Nov 2010-Jan 2011	23.3 (22,24)	Right^1,2^	Sitting^3,4^	Oscillometric device	Omron i-c10	22–42 cm	not relevant
E	305	May-Jun 2011	23.0 (16,29)	Right^1^	Sitting	Oscillometric device	Omron i-c10	22–42 cm	not relevant
F	131	Nov 2010-Feb 2011	23.2 (22,25)	Right^1,2^	Sitting^3,4^	Oscillometric device	Omron i-c10	22–42 cm, > 42 cm	not relevant
G	1302	Oct-Dec 2010	#	Right^1^	Sitting	Oscillometric device	Omron M6	17–22 cm, 22–32 cm, 32–42 cm	not relevant
H	137	May-Jul 2010	#	Right^1,2^	Sitting^3^	Oscillometric device	Omron M6	22–32 cm	not relevant
I	168	Jan-Mar 2011	21.3 (16,25)	Right^1,2^	Sitting^3,4^	Oscillometric device	Omron 705IT	≤21 cm, 22–31 cm, ≥ 32 cm	not relevant
J	922	Jan-May 2011	19.4 (12,25)	Right^1^	Sitting	Oscillometric device	Omron HEM-907	17–22 cm, 22–32 cm, 32–42 cm	not relevant
K	190	Jun-Jul 2011	23.4 (19,32)	Right^1^	Sitting^3^	Oscillometric device	Datascope Accutorr Plus	13–20 cm, 21–27 cm, 28–35 cm, 36–46 cm	not relevant
L	162	Nov-Dec 2010	#	Right^1^	Sitting	Oscillometric device	Citizen CH-432B	20–26 cm, 25–34 cm, 32–43 cm	not relevant

# Room temperature not measured, ^1^If the measurement was done on the left arm that was recorded, ^2^The reason for use of left arm was also recorded, ^3^If the measurement was done in the supine posture, that was recorded, ^4^The reason why the measurement was done in the supine posture was also recorded.

The instructions to record the activities and behaviour of the participant before the examination was added to the EHES protocol after most of the pilot surveys had already started. Therefore, in only five surveys were some of the participant’s activities (smoking, physical activity, meals and drinking) before the examination recorded.

In all surveys, three blood pressure measurements were taken in a sitting posture from the right arm. In most surveys, occasional deviations for medical reasons were recorded. (Table 2) During the site visits, it was observed that the measurers sometimes forgot to check the position of the subject, resulting in an unsupported back and/or arm, or crossed legs, or the subjects were talking during or between the measurements. After these were pointed out, the problems were usually corrected immediately and special attention by the survey organizers was paid to these issues during the rest of the survey.

The simple mercury sphygmomanometer was used in three surveys and oscillometric devices in nine. For oscillometric devices, six different models from three manufacturers were used. Each model of the oscillometric devices had passed validation following the British Hypertension Society protocol [13], International protocol [14] or Advancement of Medical Instrumentation (AAMI) protocol [15] (Table 2).

In nine out of 12 surveys, more than one cuff size was available (Table 2). Arm circumference was measured in ten surveys (Table 3). When comparing measured arm circumferences to the size of the cuff used for the measurement, the miss-cuffing (use of too small or too large cuff) was observed only in 1–5% of the subjects, except in one survey where only one cuff was available and 20% of subjects were miss-cuffed (Table 3). In three surveys which did not measure arm circumference, the occurrence of miss-cuffing would have been more likely to happen, especially if the used cuffs did not have markings to indicate correctness of the cuffs for the specific arm circumference. In all these three surveys, cuffs with indicators to assess the correctness of the cuff size were used.

**Table 3:** **Recording of cuff size used and measured arm circumference, mean arm circumference, proportion of miss-cuffed subject, and proportion of identical readings**

**Pilot survey**	**Recorded cuff size used**	**Arm circumference measured and recorded (M = measured, not recorded, B = measured and recorded, N = Not measured)**	**Mean arm circumference (min,max)**	**Proportion of miss-cuffed subject (based on optimal arm circumference reported on the cuff)**	**Proportion of identical readings between 1st and 2nd measurement**		**Proportion of identical readings between 2nd and 3rd measurement**	
					**SBP**	**DBP**	**SBP**	**DBP**
A	No	B	30.8 cm (24.5,43.5)	#	8%	29%	16%	28%
B	Yes	B	31.6 cm (23.0,48.0)	1%	10%	29%	31%	41%
C	Yes	B	28.3 cm (20.0, 40.0)	0%	10%	13%	19%	19%
D	Yes	N	§	#	4%	10%	7%	12%
E	Yes	B	29.9 cm (22.0,41.5)	0%	5%	9%	6%	11%
F	Yes	B	28.9 cm (21.0,41.5)	2%	3%	9%	9%	11%
G	Yes	M	§	#	7%	12%	8%	13%
H	Yes	B	30.1 cm (21.0,48.0)	20%	3%	8%	5%	9%
I	Yes	B	30.9 cm (22.0,46.0)	0%	8%	10%	8%	10%
J	Yes	N	§	#	6%	14%	9%	15%
K	Yes	B	30.4 cm (21.5,43.3)	5%	4%	10%	7%	10%
L	Yes	B	30.4 cm (24.0,40.0)	1%	5%	9%	5%	11%

§ Arm circumference not measured, # Not possible to calculate.

The proportion of identical sequential measurements was lower between the first and the second measurement than between the second and the third measurement for both systolic and diastolic blood pressure in three surveys using simple mercury sphygmomanometers. In each survey, the proportion of identical readings was higher for diastolic than for systolic blood pressure. Overall, the proportion of identical sequential measurements was high only in two surveys (28% or over) using simple mercury sphygmomanometer (Table 3).

Terminal digit preference was not a problem when oscillometric devices were used. In one of the three surveys which used mercury sphygmomanometers, a clear terminal digit preference for zero was seen for both systolic and diastolic blood pressure (Tables 4 and 5).

**Table 4:** **Proportion of terminal digits for systolic blood pressure**

**Device**	**Survey**	**Terminal digit (%)**										**p-value for** ***χ*** ^**2**^ **-test**
		**0**	**1**	**2**	**3**	**4**	**5**	**6**	**7**	**8**	**9**	
Mercury sphygmomanometer	A	22	0	23	0	21	0	18	0	17	0	0.4488
	B	33	6	9	3	5	22	5	4	9	4	<0.0001
	C	18	0	20	0	20	0	19	0	23	0	0.1352
Omron i-C10	D	9	11	11	9	10	11	11	9	11	9	0.9826
	E	11	10	10	9	10	10	9	11	10	9	0.7936
	F	12	9	11	7	11	7	6	13	15	9	0.0007
Omron M6	G	11	10	9	10	11	10	10	10	9	10	0.0464
	H	10	8	12	8	9	11	9	13	12	8	0.2126
Other Omron models	I	10	8	12	9	11	8	9	9	11	12	0.4071
	J	11	10	12	10	9	9	9	10	10	10	0.1308
Other brands	K	9	11	9	12	11	10	13	7	9	9	0.1753
	L	10	10	13	10	9	10	8	9	9	11	0.5258

**Table 5:** **Proportion of terminal digits for diastolic blood pressure**

**Device**	**Survey**	**Terminal digit (%)**										**p-value for** ***χ*** ^**2**^ **-test**
		**0**	**1**	**2**	**3**	**4**	**5**	**6**	**7**	**8**	**9**	
Mercury sphygmomanometer	A	21	0	19	0	26	0	18	0	16	0	0.0392
	B	35	8	8	5	7	17	6	5	6	4	<0.0001
	C	22	0	17	0	19	0	19	0	23	0	0.0317
Omron i-C10	D	15	12	9	9	8	9	10	10	8	9	0.0122
	E	11	9	9	9	11	10	9	12	10	11	0.2596
	F	11	10	7	10	9	12	11	10	9	10	0.4936
Omron M6	G	10	9	10	10	10	10	10	10	10	10	0.7859
	H	9	9	10	12	10	10	10	11	10	9	0.9850
Other Omron models	I	12	12	10	11	12	9	9	9	9	8	0.1123
	J	11	9	10	11	9	10	10	11	10	9	0.4148
Other brands	K	11	11	10	12	9	10	8	8	9	12	0.2285
	L	11	9	11	9	11	9	9	11	11	9	0.9030

## Discussion

Reliable population level information on blood pressure levels and prevalence of hypertension are needed for developing evidence based policy and planning prevention activities as well as for research. Obtaining such information is challenging, since the measurement environment, behaviour of the subject, measurement protocol, device used for the measurements and the observers can each affect the accuracy of the blood pressure measurements.

To overcome the challenges, the EHES Pilot Project prepared a European level standardized protocol for blood pressure measurement, a training programme and quality control to support the collection of high quality and comparable blood pressure data across populations. The protocol was tested in 12 pilot surveys with encouraging results.

In general, the room temperature could be standardized to a comfortable level and it was possible to eliminate outside disturbance. This is easiest when examinations are carried out in existing health care facilities designed for medical examinations or in a mobile unit especially designed for this purpose. When temporary examination clinics are set up for the survey, it may be difficult to find suitable locations which are available for a relatively short period of time. It is more difficult to standardize the measurement environment when the measurements are conducted at the participants’ home. In this case, the survey organizers cannot control the room temperature or possible disturbances during the measurement. Therefore it is particularly important to record the room temperature and possible disturbances during the measurement.

Controlling the behaviour of the participant before the examination requires clear instructions for the survey invitees. The person welcoming the participant to the examination centre or staff taking the blood pressure measurements should check that the participant does not have an uncomfortably full bladder before the blood pressure measurement begins. Information on activities before measurement should be recorded.

The importance of standardized measurement procedures in multicentre and international studies has been reported [12,34]. The EHES Pilot Project demonstrated that a standardized protocol is a feasible tool for minimizing variation due to measurement technique. In addition, the measurers must be trained properly. This has been shown by the National Health and Nutrition Examination Survey (NHANES) of the United States [35] and the WHO MONICA Project [36], where extensive training has been found to be crucial for the success of standardization. Similar results have been reported from hypertension studies [34,37].

To evaluate the use of a standardized protocol in the field, monitoring is required during the fieldwork. These monitoring visits should be done on a regular basis to observe how fieldwork teams conduct measurements, how they interact with survey invitees and also how they interact with each other. The interval between monitoring visits depends on the duration of the fieldwork. When the fieldwork is conducted within a few months, at least one monitoring visit should be done at the beginning of the fieldwork and one during the remaining fieldwork period. In surveys with a longer fieldwork period, the interval of the monitoring visits should be conducted so that they take into account changes of examination sites and also possible changes in the fieldwork personnel. Depending on the survey, these visits can be every 2–4 months. Also, re-training or refresher sessions, during which standardized measurement protocols are gone through and measurements are conducted under supervision, are needed to maintain the level of standardization, especially if the fieldwork last several months or years.

Traditionally, the mercury sphygmomanometer has been considered as the ‘gold standard’ for blood pressure measurement. In Europe, the use of mercury sphygmomanometers in HESs has declined during the past decades. In future, all European HESs will have to use devices other than mercury sphygmomanometers, as the EU regulation 847/2012 banned the sale of mercury sphygmomanometers from 10 April 2014 onwards [38]. It has been shown that changing from mercury sphygmomanometers to non-mercury devices (oscillometric or aneroid devices) affects the monitoring of blood pressure levels and hypertension prevalence [39]. With changing devices, it is important to conduct validation studies to obtain calibration estimates to ensure comparability of results within and between populations.

A variety of oscillometric devices for blood pressure measurement is on the market. Many of them have passed at least one of the validation tests based on the British Hypertension Society protocol [13], International protocol of the European Society of Hypertension [14] or Association for the Advancement of Medical Instrumentation (AAMI) protocol [15]. These validation protocols are designed to evaluate the accuracy of the device for clinical practice. However, the allowed deviations are too large for epidemiological studies. The information about the devices which have passed the validation can be found for example on the British Hypertension Society web site at http://www.bhsoc.org/bp-monitors/bp-monitors and the dabl® Educational Trust web site at http://www.dableducational.org/sphygmomanometers.html. Comparison of the different oscillometric devices is difficult, since their algorithms used for determination of the systolic and diastolic blood pressure are not openly accessible and these are likely to change over time as the measurement devices are improved. This also makes it difficult to replicate the validation studies for the older models. Therefore, the auscultation method with calibrated devices will remain superior and should be considered as ‘gold standard’ also in future. For the auscultation method, there are validated alternatives to the mercury sphygmomanometer [40,41].

The importance of correct cuff size for the accuracy of the blood pressure measurement has been emphasized. Use of too large a cuff can result up to 10–30 mmHg lower systolic and diastolic blood pressure readings and on the other hand, use of too small a cuff to 3–12 mmHg higher systolic and 2–8 mmHg higher diastolic blood pressure readings [11]. Regarding cuff size, the width of the cuff bladder should be at least 40% of the arm circumference and the length at least 80% to minimize the error due to incorrect cuff size [42]. As shown by the EHES Pilot results, there is a large variety of cuff sizes on the market. Each manufacturer has its own cuffs. Some manufacturers have provided so called ‘universal’ cuffs, which should be used for a wide range of arm circumferences (22–42 cm). Variation in cuff sizes should not be a problem as long as the manufacturers provide several different cuff sizes and the appropriate cuff size is used for each participant.

Miss-cuffing was not a major problem in the EHES pilot. Some countries encountered problems in obtaining more than one cuff size (universal cuff) for their oscillometric device through their national supplier.

Observer error can be minimized when oscillometric devices are used. For mercury sphygmomanometers, minimizing the observer error requires thorough practical training [43,44].

The final decision on the blood pressure measurement protocol to be used in national HES in Europe is made by each country. The actual deviations between protocols used were small and could be easily corrected; in most cases, differences were only in the devices used. These and other possible deviations were usually caused by the need to follow trends from past surveys within the country. Nevertheless, there is a shared desire for comparability of the blood pressure measurement results between countries. This can be obtained through a joint protocol. The EHES blood pressure measurement protocol was followed well in all 12 EHES pilot surveys, and the external quality control helped to minimize observer errors. In the countries which had an on-going full-size national HES, the EHES protocol was also followed well. These surveys had based their blood pressure measurement protocols for previous European level standards of the European Health Risk Monitoring (EHRM) Project, which was the base for the EHES protocol [45].

## Conclusions

Our experience from the EHES Pilot Project is in line with the results of previous studies showing that blood pressure measurements can be standardized across countries and over time. This requires a detailed, standardized measurement protocol; adequate training; and monitoring during the fieldwork as well as retrospective quality assessment. However, the recent EU regulation banning the sale of the mercury sphygmomanometers in European Union Member States [38] and the differences between the oscillometric devices have set new challenges for the standardization of blood pressure measurement.

## Appendix 1 Ethical committees providing the approval for the surveys

**Czech Republic:** Ethical Committee of the National Institute of Public Health, Prague.

**Finland:** Helsingin ja Uudenmaan sairaanhoitopiiri, Koordinoiva eettinen toimikunta, Helsinki.

**Germany:** Ethik Kommission, Charité, Universitätsmedizin, Berlin.

**Greece:** Ethical Committee of Hellenic Health Foundation, Athens.

**Italy:** Ethical Committee of the Istituto Superiore di Sanità, Rome.

**Malta:** Health Ethics Committee, Department of Health Information & Research.

**Netherlands:** Medisch Ethische Toetsingscommissie, Universitair Medisch Centrum, Utrecht.

**Norway:** The Regional Committee for Medical and Health Research Ethics REK North.

**Poland:** Terenowej Komisji Bioetycznej przy Instytucie Kardiologii.

**Portugal:** Comissão de Ética, Instituto Nacional de Saúde, Doctor Ricardo Jorge.

**Slovakia:** Ethical Committee, Regional Authority of Public Health (RAPH), Banská Bystrica.

**UK/England:** Oxfordshire REC A, National Research Ethics Service, NHS.

## Appendix 2 Sites and key personnel contributing to the EHES Pilot Project

**Czech Republic:** National Institute of Public Health, Prague: Ruzena Kubinova, Nada Capkova, Jana Kratenova and Michala Lustigova.

**Finland:** National Institute for Health and Welfare (THL). EHES Reference Centre: Kari Kuulasmaa, Hanna Tolonen, Katri Kilpeläinen, Päivikki Koponen, Sanna Ahonen, Johanna Mäki-Opas, Ari Haukijärvi, Tarja Palosaari, Georg Alfthan, Jari Kirsilä.

National pilot survey: Satu Männistö, Katja Borodulin, Liisa Saarikoski, Anne Juolevi, Markku Peltonen, Tiina Laatikainen, Erkki Vartiainen, Jouko Sundvall, Laura Lund, Antti Jula, Eija Purkamo.

**Germany:** Robert Koch Institute, Berlin. For the DEGS Study Team: Antje Gösswald, Cornelia Lange, Panagiotis Kamtsiuris.

**Greece:** Hellenic Health Foundation, Athens. Antonia Trichopoulou, Valentini Konstantinidou, Androniki Naska, Dimosthenis Zilis, Vardis Dilis, George Adarakis, Ioulia Goufa, Georgia Stasinopoulou, Elisabeth Valanou, Perikles Karathanasis, Nikolaos Bilalis, Philippos Orfanos, Tina Karapetyan, Despina Oikonomidou, Eirini Frangogeorgi and Konstantinos Mine.

**Italy:** Istituto Superiore di Sanità, Rome. EHES Reference Centre: Susanna Conti, Mark Kanieff.

National Pilot Survey: Luigi Palmieri, Chiara Donfrancesco, Cinzia Lo Noce, Francesco Dima, Amalia De Curtis, Licia Iacoviello, Diego Vanuzzo, Simona Giampaoli.

**Malta:** Department of Health Information & Research, Gwardamangia: Neville Calleja, Dorothy Gauci.

**The Netherlands:** National Institute of Public Health and the Environment (RIVM), Bilthoven: W.M. Monique Verschuren.

**Norway:** Norwegian Institute of Public Health: Grethe S. Tell, Patricia Schreuder, Sidsel Graff-Iversen, Nina Hovland; University of Bergen: Kristin Klock.

Statistics Norway. EHES Reference Centre: Johan Heldal, Susie Jentoft.

**Poland:** The Cardinal Stefan Wyszynski Institute of Cardiology, Warsaw: Grażyna Broda, Aleksandra Piwonska, Jerzy Piwoński, Paweł Kurjata, Walerian Piotrowski, Maria Polakowska, Anna Waśkiewicz, Elzbieta Sygnowska.

**Portugal:** Instituto Nacional de Saúde Dr. Ricardo Jorge, Lisbon: Carlos Dias, Ana Paola Gil.

**Slovakia:** Regional Authority of Public Health, Banská Bystrica. Maria Avdicova, Katarina Francisciova, Jana Namesna, Silvia Kontrosova.

**UK:** UCL (University College London), London: Jennifer Mindell, Nicola Shelton, Barbara Carter-Szatynska, Alison Moody.

Health and Social Care Information Centre, London: Rachel Craig, Susan Nunn, Deanna Pickup, Chloe Robinson.

The NHS Information Centre: Steve Webster, Victoria Cooper.
